# SNPTracker: A Swift Tool for Comprehensive Tracking and Unifying dbSNP rs IDs and Genomic Coordinates of Massive Sequence Variants

**DOI:** 10.1534/g3.115.021832

**Published:** 2015-11-19

**Authors:** Jia-En Deng, Pak C. Sham, Miao-Xin Li

**Affiliations:** * Department of Psychiatry, University of Hong Kong; † Centre for Genomic Sciences, University of Hong Kong; ‡ State Key Laboratory for Cognitive and Brain Sciences, University of Hong Kong; § Center for Reproduction, Development and Growth, University of Hong Kong, Hong Kong SAR, China.

**Keywords:** single nucleotide polymorphism (SNP), dbSNP, RefSnp (rs) ID, genomic coordinate, sequence variant

## Abstract

The reference single nucleotide polymorphism (rs) ID in dbSNP (http://www.ncbi.nlm.nih.gov/SNP/) is a key resource identifier, which is widely used in human genetics and genomics studies. However, its application is often complicated by the varied IDs of different versions. Here, we developed a user-friendly tool, SNPTracker, for comprehensively tracking and unifying the rs IDs and genomic coordinates of massive sequence variants at a time. It worked perfectly, and had much higher accuracy and capacity than two alternative utilities in our proof-of-principle examples. SNPTracker will greatly facilitate genetic data exchange and integration in the postgenome-wide association study era.

The dbSNP database (http://www.ncbi.nlm.nih.gov/SNP/) is a standard resource for genetic and genomic studies in which the sequence variants, most of which are single nucleotide polymorphisms (SNPs), are identified by RefSnp (rs) ID number (Sherry *et al.* 1999). The rs ID system provides a very convenient way for geneticists to exchange data and knowledge. However, the dbSNP rs IDs of sequence variants were updated frequently by merging redundant ones and removing falsely-discovered ones. This is particularly true when high throughput genotyping technologies are being used widely (Kitts *et al.* 2014). So a sequence variant may have multiple different rs IDs in different bioinformatics databases and genetics datasets. This issue is complicating annotation, data merging and exchange of sequence variants in genetic and genomic analyses.

To our knowledge, there have been two tools for updating the rs IDs of sequence variants by batch, LiftRsNumber (Zhan 2011) and dbSNP Batch Query (2005). LiftRsNumber was developed to convert dbSNP rs IDs over different versions for genetic analyses in 2011. It should be noted that dbSNP has enlarged substantially in the past 4 years, during which many sequence variants were merged and deleted once or multiple times. Because LiftRsNumber has not been updated since 2011, it may not adequately consider the emerging complexity of the rs ID system. In addition, as LiftRsNumber was originally an internal tool that used resources only from its developers’ server, users have to modify its source codes to adopt new resource datasets. This is inconvenient and difficult for many users. In contrast, dbSNP Batch Query (2005) is a convenient online tool. However, it cannot process a batch with over 30,000 SNPs, making it infeasible for a genome-wide association study (GWAS), or whole genome sequencing dataset. Besides, it does not output data in a format recognizable by a popular genetic analysis tool (*e.g.*, PLINK; Purcell *et al.* 2007), and cannot flexibly retrieve rs IDs by genomic coordinates. So, dbSNP Batch Query (2005) is actually far from convenient in many scenarios.

In the present study, we developed an accurate and convenient standalone utility, named SNPTracker, for efficiently tracking and unifying dbSNP rs IDs and genomic coordinates of massive sequence variants for further genetic and genomic analyses. We also compared the accuracy and coverage of SNPTracker with those of LiftRsNumber (Accessed in April 2015) and dbSNP Batch Query (Accessed in April 2015).

## Materials and Methods

SNPTracker is built upon an algorithm for comprehensively converting the previous rs IDs into the latest ones by using three dbSNP resource datasets, RsMergeArch, SNPHistory and SNPChrPosOnRef from dbSNP (Sherry *et al.* 1999). The RsMergeArch dataset records all SNP merging events of rs IDs in dbSNP. It contains three types of rs IDs of a variant, the early version ID (rsLow), the later version ID (rsHigh), and the current ID (rsCurrent). In a merging event, dbSNP always merges the rsHigh ID to the rsLow one. The SNPHistory dataset contains variants that have been deleted in dbSNP. The SNPChrPosOnRef dataset includes chromosomes and genomic coordinates of all known variants in dbSNP. The process is separated into two main parts, data preparation and ID mapping (Supporting Information, Figure S1).

### Tracking by rs IDs

SNPTracker starts with a procedure to extract effective data for all input sequence variants from the three aforementioned resource datasets. The merged events of all input variants are read from the RsMergeArch dataset at first. As some variants were merged several times in dbSNP, SNPTracker traces such variants from the first merge event to the latest one by walking through intermediate rs IDs. For some variants, submitters withdrew rs IDs as descripted in the dbSNP Guide document (http://www.ncbi.nlm.nih.gov/books/NBK3861/). Therefore, SNPTracker next reads the SNPHistory dataset and marks the latest rs IDs, which have been completely deleted. However, one may also resubmit his or her withdrawn variants, probably because the deleted variants are widely used by other users. These resubmitted variants are marked by “reactivation” in the SNPHistory dataset. Accordingly, SNPTracker also uses these reactivated rs IDs for tracking. At the end of the data preparation procedure, SNPTracker reads the SNPChrPosOnRef dataset to retrieve coordinates for all input variants. Note that some variants have problematic coordinates, such as being mapped to multiple contigs (Multi) and not being mapped to any current chromosome (NotOn). SNPTracker marks variants with these annotations as well. Finally, input variants not existing in the SNPChrPosOnRef dataset but in the SNPHistory dataset are marked as being deleted. After preparing the effective data, SNPTracker then goes through every input variant to update its rs ID and coordinate. The extracted rs IDs and annotations are stored in a Hash Table. For variants with valid rs IDs, SNPTracker reports the latest rs IDs and genomic coordinates into a results file. In contrast, SNPTracker reports the rs IDs with special codes in a separate error file. Besides, the rs IDs marked as being deleted will be also saved into the error file.

### Tracking by genomic coordinates

SNPTracker can also track the rs IDs by variants’ genomic coordinates. The input genomic coordinates are utilized to retrieve the rs IDs from the SNPChrPosOnRef dataset first. SNPTracker assumes the input coordinates are 1-based. Moreover, it also automatically corrects for 0-based coordinates. Given the rs IDs, SNPTracker will follow the above procedure of tracking by rs IDs. In dbSNP, the SNPChrPosOnRef dataset is available only for reference genomes, hg19 and hg38. If the input coordinates are hg17 and hg18, SNPTracker first automatically converts the coordinates into hg19 by the UCSC LiftOver algorithm, and then retrieves the rs IDs by the new coordinates.

### Tracking by a map/bim file

To facilitate genetic analysis in PLINK (Purcell *et al.* 2007), SNPTracker also has a function for updating rs IDs for variants in marker files in the “bim” or “map” formats. SNPTracker reads the rs IDs (if available) or genomic coordinates first. Given the rs IDs or genomic coordinates, the main procedures involved are the same as aforementioned. Users can straightforwardly use the output of SNPTracker as one of the inputs of PLINK for further analyses.

### Compiling raw resource data from dbSNP

To speed-up the tracking procedure, SNPTracker uses a compiled resource dataset from the original dbSNP resource data. In the SNPTracker resource file, all previous versions of rs IDs in the dbSNP RsMergeArch dataset are directly mapped to the current version of rs IDs so that SNPTracker can quickly retrieve the current version of rs IDs for any version of rs IDs. This is done only once by an iterative algorithm in SNPTracker when there is a new dbSNP resource dataset. The complied resource data of the latest version of dbSNP have been provided on the website of SNPTracker, and can be automatically downloaded by SNPTracker. Meanwhile, users are also able to use the original resource datasets directly downloaded from dbSNP. SNPTracker will then automatically compile the resource datasets prior to the actual tracking procedure (see detail in the online user-manual http://grass.cgs.hku.hk/limx/snptracker/).

### Data availability

SNPTracker is freely available for download at http://grass.cgs.hku.hk/limx/snptracker/.

## Results

### Usage of SNPTracker

SNPTracker encoded by Java (https://www.java.com) has a user-friendly command-line interface for tracking rs IDs in either small- or large-scale datasets. Once initiated for the first time, SNPTracker automatically downloads the resource datasets of Human Build 142 from the SNPTracker server in parallel. The input is either rs IDs or coordinates of variants stored in a text file. The simplest way to run SNPTracker is by its default model with the input file path and prefix of output file name:

java -jar snptracker.jar input.txt output,

in which the first column of the input file is the rs ID column, and the version of output coordinate is hg19 by default. Besides, users could also flexibly specify the input/output settings in three different ways (including the conventional map/bim files recognizable for PLINK, see detail in the online user-manual, http://grass.cgs.hku.hk/limx/snptracker/).

Moreover, we also provided a web-based front end to further ease the usage of SNPTracker for relatively small-scale datasets (http://grass.cgs.hku.hk/limx/snptracker/online/online.html). On the webpage, users can specify the settings by clicking buttons, and submit a SNP tracking task to our web server. Once the result is available, a downloading link for the result will be sent to users by E-mail.

### SNPTracker updated more rs IDs than LiftRsNumber

We first asked whether SNPTracker updates the latest version of rs IDs better than LiftRsNumber. This comparison was based on an old in-house GWAS dataset (genotyped by Illumina Human610-Quad BeadChip and Illumina Human550-Quad Beadchip) that contains 4,801,761 SNPs on a workstation with 6 GB RAM and i5-3570 3.40GHz CPU. Both SNPTracker and LiftRsNumber were used to convert the rs IDs in the dataset to dbSNP Build 142, and map variants’ coordinates onto the hg38 human reference genome. As LiftRsNumber was hard-coded to use the resources of the old dbSNPs, we manually modified its source codes to allow it to read the latest dbSNP resource datasets. SNPTracker successfully updated 25,591 more rs IDs than LiftRsNumber in total (Table 1). Most of these SNPs were reported to have unknown and deleted rs IDs by LiftRsNumber. In contrast, SNPTracker successfully traced back rs IDs at all of these SNPs except for 119 deleted and 26 invalid SNPs (Table 1). Supposedly, LiftRsNumber only used the current rs ID in the RsMergeArch dataset to track rs IDs, which may have no information for some SNPs. However, SNPTracker also used the previous rs IDs in the RsMergeArch dataset to track SNP IDs (detailed in *Methods*). Besides, SNPTracker produced more detailed annotation about the problematic SNPs (Table 1). For instance, SNPTracker reported 572 and 15 SNPs that were no longer mapped to any current chromosomes (NotOn), and coordinates according to dbSNP Build142 in the old GWAS dataset.

**Table 1:** Comparison between SNPTracker and LiftRsNumber

		SNPTracker	LiftRsNumber
	Unchanged	4,749,300	4,749,733
	Merged	51,725	26,432
Problematic*^a^*	Unknown rs ID	0	25,591
	Deleted	119	5
	Invalid	26	—
	Chr_NotOn	572	—
	No coord˙	15	—
	Duplicated rs ID	4	—
Total		4,801,761	4,801,761

a Notes: “Invalid” denotes a variant which has an invalid rs ID because submitters withdrew and merged SNP clusters. chr_NotOn denotes a variant which is no longer mapped to any current chromosome in SNPChrPosOnRef dataset. No coord˙ denotes a variant mapped to multiple contigs or a contig without exact coordinates information in SNPChrPosOnRef. Duplicated rs IDs denotes that the current version ID and the early version ID of a same variant were both in the query text file.

We also analyzed three old Affymetrix genotyping platforms (GeneChip 100K Array Set, GeneChip Mapping 500K Array Set, and GenomeWide Array Set), and 3 other Illumina genotyping platforms (HumanHap 300-Duo Array Set, HumanHap 550-Duo Array Set, and HumanHap 650Y Array Set) to evaluate the performance of SNPTracker. The Affymetrix GeneChip Mapping 500K Array Set included two subarrays, 250K Nsp Array (containing 262,264 variants), and 250K Sty Array (containing 238,304 variants). Both SNPTracker and LiftRsNumber were used to convert the rs IDs in both datasets to dbSNP Build 142, and mapped variants’ coordinates onto the hg19 human reference genome. For variants with rs ID in the 250K Nsp Array, SNPTracker successfully converted the rs ID of nine more variants that were reported to have unknown ID by LiftRsNumber, and reported 31 variants that were no longer mapped to any current chromosomes but were not recognized by LiftRsNumber (Table S1). Moreover, SNPTracker also successfully retrieved the rs IDs for 4383 variants by their coordinates (hg17), which had no rs ID in the 250K Nsp Array. In contrast, LiftRsNumber cannot trace rs IDs by coordinates, and the 4383 variants were reported as unchanged rs IDs with “—”. Furthermore, SNPTracker gave more detailed annotation at 234 problematic SNPs and rs IDs (Table S1). The pattern of results was quite similar for 250K Sty Array (Table S2). The Affymetrix GeneChip 100K Array Set, Affymetrix GenomeWide Array Set, Illumina HumanHap 300-Duo Array Set, Illumina HumanHap 550-Duo Array Set, and Illumina HumanHap 650Y Array Set contained 116,204, 440,794, 318,223, 561,436 and 660,876 variants, respectively. The comparison results of these five platforms between SNPTracker and LiftRsNumber are shown in Table S3, Table S4, Table S5, Table S6, and Table S7. In summary, SNPTracker has three advantageous features: (i) SNPTracker can trace variants without rs IDs through coordinates; (ii) SNPTracker can produce more detailed annotation for problematic variants; (iii) SNPTracker can trace more variants that were reported to have unknown IDs by LiftRsNumber;

### SNPTracker produced more rs IDs than dbSNP Batch

We also made a comparison between SNPTracker and dbSNP Batch Query (2005). Since dbSNP Batch is limited to 30,000 rs IDs at each query, we randomly drew 30,000 rs IDs from the above-mentioned in-house GWAS dataset for comparison. These rs IDs were mapped onto dbSNP Build 142 with coordinates of hg38. While both tools produced very similar number of rs IDs, SNPTracker had 29,824 (out of 29,999) consistent results with dbSNP Batch. In addition, SNPTracker had 175 more converted SNPs than dbSNP Batch Query (2005), 29,999 *vs.* 29,824 respectively. We then manually retrieved the rs IDs for the 175 variants via the dbSNP’s individual SNP search webpage (http://www.ncbi.nlm.nih.gov/SNP/) one by one. It turned out that all of the rs IDs of the 175 variants are consistent with those given by SNPTracker, suggesting SNPTracker retrieved the rs IDs even more comprehensively than dbSNP Batch Query (2005). For example, dbSNP Batch failed to return any rs ID for rs115610697. Both SNPTracker and the dbSNP’s individual SNP search webpage reported this rs ID was merged into rs113010839. A possible reason why the 175 rs IDs was ignored by dbSNP Batch Query (2005) is that it may ignore some of the previous rs IDs that are deleted after merging into the current rs IDs.

### Conclusions

In summary, SNPTracker is a user-friendly tool for comprehensively tracking and unifying rs IDs and genomic coordinates of massive amounts of sequence variants. It has a higher success rate in updating rs IDs than the alternative tools, and can export the results in formats recognizable by popular genetics analysis tools. SNPTracker will play a very useful role in facilitating genetic data exchange and integration in GWAS.
